# The functional activity and effective connectivity of pulvinar are modulated by individual differences in threat-related attentional bias

**DOI:** 10.1038/srep34777

**Published:** 2016-10-05

**Authors:** Yuko Hakamata, Eisuke Sato, Shotaro Komi, Yoshiya Moriguchi, Shuhei Izawa, Norio Murayama, Takashi Hanakawa, Yusuke Inoue, Hirokuni Tagaya

**Affiliations:** 1 Department of Adult Mental Health, National Institute of Mental Health, National Center of Neurology and Psychiatry, Tokyo, Japan; 2 Department of Health Sciences, Kitasato University School of Allied Health Sciences, Kanagawa, Japan; 3 Department of Clinical Psychology, Graduate School of Education, The University of Tokyo, Tokyo, Japan; 4 Department of Medical Radiological Technology, Kyorin University School of Health Sciences, Tokyo, Japan; 5 Department of Clinical Engineering, Kitasato University School of Allied Health Sciences, Kanagawa, Japan; 6 Integrative Brain Imaging Center, National Center of Neurology and Psychiatry, Tokyo, Japan; 7 Department of Health Administration and Psychosocial Factor Research Group, National Institute of Occupational Safety and Health, Kanagawa, Japan; 8 Department of Diagnostic Radiology, Kitasato University School of Medicine, Kanagawa, Japan.

## Abstract

The pulvinar is important in selective attention, particularly to visual stimuli under the focus of attention. However, the pulvinar is assumed to process emotional stimuli even outside the focus of attention, because of its tight connection with the amygdala. We therefore investigated how unattended emotional stimuli affect the pulvinar and its effective connectivity (EC) while considering individual differences in selective attention. fMRI in 41 healthy human subjects revealed that the amygdala, but not the pulvinar, more strongly responded to unattended fearful faces than to unattended neutral faces (UF > UN), although we observed greater EC from the pulvinar to the amygdala. Interestingly, individuals with biased attention toward threat (i.e., attentional bias) showed significantly increased activity (UF > UN) and reduced grey matter volume in the pulvinar. These individuals also exhibited stronger EC from the pulvinar to the attention-related frontoparietal network (FPN), whereas individuals with greater attentional control showed more enhanced EC from the pulvinar to the amygdala, but not the FPN (UF > UN). The pulvinar may filter unattended emotional stimuli whose sensitivity depends on individual threat-related attentional bias. The connectivity patterns of the pulvinar may thus be determined based on individual differences in threat-related attentional bias and attentional control.

## Introduction

It is evolutionarily important for organisms to monitor environmental changes, such as the abrupt emergence of a potential threat, and to promptly detect significant stimuli while filtering out distractors. Such a function is traditionally discussed in the context of attention, particularly selective attention, and its sensitivity varies between individuals^1^,^2^. Neuroimaging research has emphasized the importance of the frontoparietal network (FPN) in selective attention, particularly in detecting salient and behaviourally-relevant environmental stimuli^3^,^4^. However, subcortical structures, such as the amygdala, are important in detecting affectively significant stimuli, specifically under conditions of unawareness^5^. Indeed, several previous studies have reported increased activity in the amygdala in response to emotional stimuli outside the focus of attention^6^,^7^,^8^. Therefore, it has been suggested that the subcortical pathway that directly conveys visual information from the pulvinar receiving direct inputs from the superior colliculus (SC) to the amygdala at an early stage of information processing exists in primates to promptly detect significant affective stimuli, bypassing top-down processing by the FPN. Nonetheless, the exact functional role of the pulvinar remains enigmatic in humans^9^.

The pulvinar is the largest nuclear mass in the primate thalamus and has enlarged in parallel with the temporal lobe^10^,^11^. It can be traditionally classified into two or three subdivisions (e.g., dorsomedial and ventrolateral; or lateral, inferior, and medial nuclei), and each has diverse connections with extensive cortical areas and subcortical structures^9^,^12^. Above all, the dorsomedial (or medial) nucleus is known to be tightly connected with the amygdala^12^,^13^,^14^. Early studies using electrophysiological recordings or chemical inactivations in monkeys have indicated that the pulvinar [particularly dorsal and medial portions of the lateral pulvinar (Pdm)] modulates visual selective attention^15^,^16^,^17^,^18^, which is important in distinguishing behaviourally-relevant stimuli from distractors^19^,^20^. Similarly, lesion studies in humans have shown impairment of visual selective attention following the pulvinar damage^21^,^22^. Despite the dearth of neuroimaging research focused on the pulvinar, a few neuroimaging studies have reported findings consistent with these early studies. For example, a functional magnetic resonance imaging (fMRI) study showed significant activation of dorsomedial pulvinar, homologous to Pdm in monkeys, during detection of luminance changes in flickering neutral stimuli, but only when these stimuli were attended to^23^. Another fMRI study demonstrated that the (entire) pulvinar exhibited stronger activation when subjects discriminated subtle differences in a neutral object’s position during attended conditions compared to unattended conditions^24^. These findings indicate that the pulvinar, or its dorsomedial part, directs selective attention to behaviourally-relevant or salient stimuli within the focused visual field while filtering out distracting information. However, previous fMRI studies on the pulvinar have exclusively used neutral stimuli and little is known about the pulvinar’s role in selective attention, particularly to emotional stimuli outside the focused visual field. Given that the subcortical pathway may be critical for emotional processing under conditions of unawareness^5^,^25^,^26^,^27^, the pulvinar may respond to emerging threats located outside the focus of attention.

As mentioned previously, there are considerable individual differences in the function of selective attention. For example, individuals with poorer attentional control often have difficulty in maintaining focused attention on an ongoing goal in the presence of distractors. Conversely, individuals with stronger threat-sensitivity, or “attentional bias” (AB), tend to quickly detect and direct selective attention to threat, even that in unattended locations^28^,^29^,^30^. In particular, AB is assumed to arise from the threat detection system of the amygdala, which competes with the top-down attentional control system (i.e., the FPN)^31^. Considering the pulvinar’s role in visual selective attention^15^,^16^,^17^,^18^,^21^,^22^, the pulvinar (or more specifically its dorsomedial portion) may allocate selective attention to an unattended threat via its connection to the amygdala, which may be influenced by individual differences in AB. In other words, pulvinar-to-amygdala effective connectivity (EC) during the processing of threats at unattended locations may be altered in individuals with greater AB.

We therefore investigated whether the pulvinar would respond to emotional faces at unattended locations, considering its EC to the amygdala, as well as individual differences in the function of selective attention. We specifically hypothesized that in comparison to unattended neutral faces (UN), when unattended fearful faces (UF) are processed, (1) the pulvinar, as well as the amygdala, would show heightened activity; (2) we would observe significant EC from the pulvinar to the amygdala, (3) individual differences in threat-related AB would modulate the activity of the pulvinar or its EC to the amygdala, and (4) dorsomedial part of the pulvinar would have specific relevance to these relationships. We also examined whether AB would predict any structural alterations using structural MRI, focusing on the pulvinar. In addition to studying threat-related AB, we investigated whether individual differences in behavioural performance regarding non-emotional attentional control would have an impact on pulvinar functional activity or structural volume. This helped us to determine if the pulvinar has a specific role in emotional attention or if it is only involved in non-emotional general attention.

## Results

### fMRI: Comparison between UF and UN

**Figure 1:**
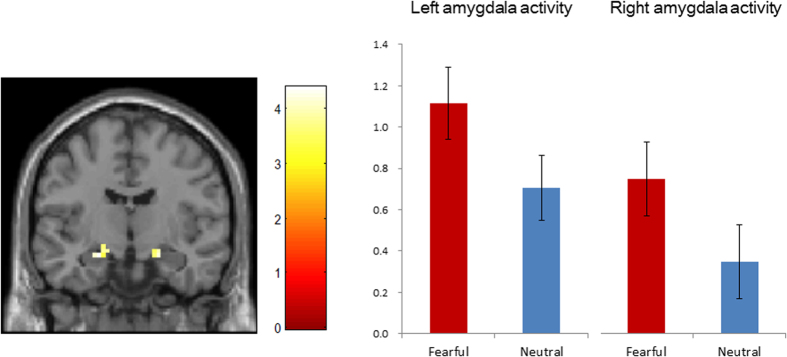
Increased activity of the bilateral amygdala upon the appearance of fearful faces vs. neutral faces outside the focus of attention. MNI coordinate: ±21 −10 −17. Significant at FWE-corrected *p* < 0.05 with small volume correction (SVC) for the left side, and at *p* < 0.001 with SVC for the right side. Colour bar indicates T-value. The coronal plane image is presented at *y* = −10.

We observed increased activity in the bilateral amygdala when fearful faces, as opposed to neutral faces, appeared in the unattended locations (i.e., UF > UN) (Table 1, Fig. 1). This finding validates that the experimental task used successfully evoked the subjects’ emotion at the neural level. Overall pulvinar activity averaged across all subjects was not significantly different between UF and UN conditions.

### Psychophysiological interaction (PPI) analysis: EC from the pulvinar to the amygdala

**Figure 2:**
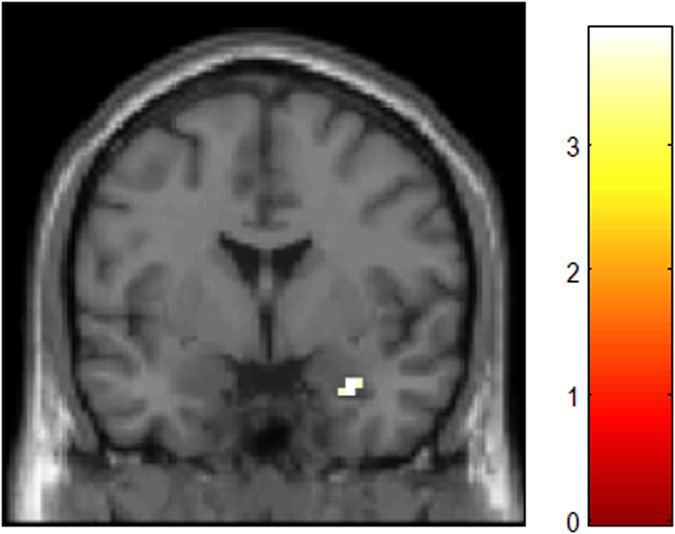
The right amygdala displays greater effective connectivity with the left pulvinar during the observation of unattended fearful faces vs. neutral faces. MNI coordinate: 30 −1 −26. Significant at FWE-corrected *p* < 0.05 with small volume correction. Colour bar indicates T-value.

**Figure 3:**
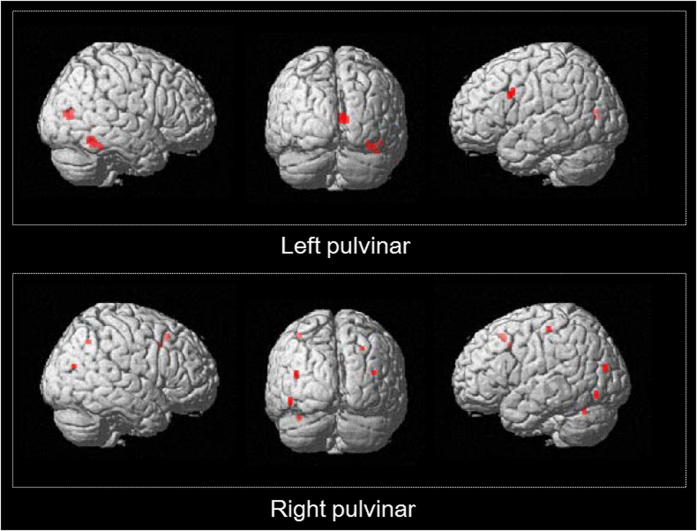
Results of the psychophysiological interaction analysis: UF < UN. Top: the left pulvinar as seed. Bottom: the right pulvinar as seed. Both sides of the pulvinar were defined by WFU PickAtlas. Significant at FWE-corrected *p* < 0.05. UF, unattended fearful faces condition; UN, unattended neutral faces condition.

Using the pulvinar defined by the Wake Forest University (WFU) PickAtlas^29^,^30^ as a seed region, PPI analysis demonstrated significantly greater EC from the left pulvinar to the right amygdala (Fig. 2) when UF were processed (as compared to UN). Moreover, the left pulvinar showed significantly greater EC to the fusiform face area (FFA) and the opercular part of the inferior frontal gyrus (OP), whereas the right pulvinar had greater EC to the Supplementary motor area (SMA), the precentral gyrus, the superior parietal lobule (SPL), and the anterior part of the midcingulate gyrus (aMCC) (Table 2, Fig. 3). Specifically, a portion including the right medial and lateral pulvinar nuclei (ml-pulvinar) were more strongly connected with the SMA and the supramarginal gyrus (SMG) (Table 2), suggesting that the pulvinar more strongly interacted with the attention-related FPN during the processing of UF than that of UN.

### Effect of individual differences in emotional and non-emotional attention on pulvinar activity and its EC

**Figure 4:**
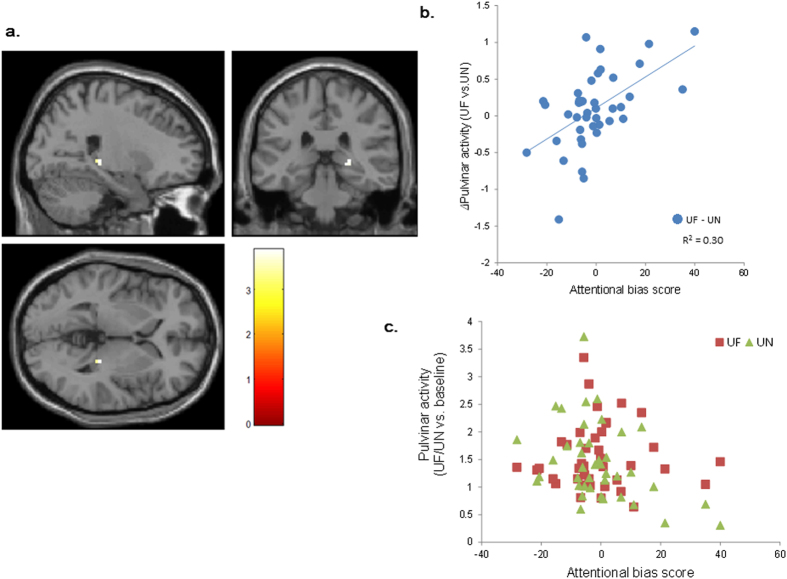
Increased activity in the right ml-pulvinar with greater attentional bias toward threat. (**a**) MNI coordinate: 24 −31 1. Colour bar indicates T-value. The figure is shown with the height threshold of *p* = 0.005 for illustration purposes. (**b**) Scatter plot of the relationship between attentional bias score and right ml-pulvinar activity in response to UF (vs. UN). (**c**) Scatter plot of the relationship between attentional bias score and right pulvinar activity in response to UF and UN. UF, unattended fearful faces condition; UN, unattended neutral faces condition; ml-pulvinar, a part of the medial and lateral pulvinar nuclei.

As a measure of individual emotional attention, AB showed a significant positive correlation with activity in the ml-pulvinar, indicating that individuals whose attention tended to be biased toward threat had increased activity in the ml-pulvinar during the processing of UF than that of UN (Table 3, Fig. 4). In contrast, indices of the Trail Making Test (TMT)^31^, a measure of non-emotional attentional control, had no correlation with functional activity in the whole brain, such that the ml-pulvinar activation was independently explained by emotional attention, but not by non-emotional attention.

**Figure 5:**
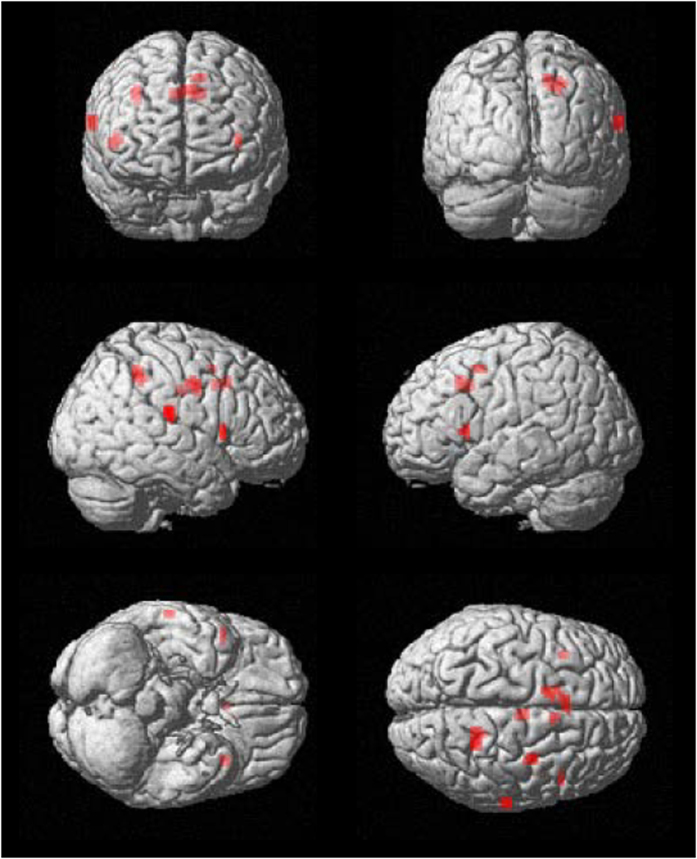
The results of PPI analysis using the right ml-pulvinar as a seed: positive correlation with attentional bias. Results were based on the PPI contrast for UF minus UN. Significant at uncorrected *p* < 0.001 and *k* < 5 (liberal threshold). PPI, psychophysiological interaction; ml-pulvinar, a part of the medial and lateral pulvinar nuclei; L, left; R, right; UF, unattended fearful faces condition; UN, unattended neutral faces condition.

In addition, AB demonstrated a significant positive relationship with the strength of the right-pulvinar seeded EC to the right postcentral gyrus (UF > UN) (Montreal Neurological Institute coordinates [MNI]: 15 −40 64; 27 mm^3^; *Z* = 4.93, familywise error [FWE]-corrected *p* < 0.05), while the left-pulvinar seeded EC was not significantly predicted by AB. Focusing on the ml-pulvinar, we observed a significant positive relationship between AB and the strength of the ml-pulvinar seeded EC to the OP, the precentral gyrus, SMG, aMCC, SMA, and the anterior insula (AI) (Table 4, Fig. 5). In other words, threat-sensitive individuals exhibited more enhanced EC from the ml-pulvinar to the FPN when the processing of UF than that of UN. Particularly, the findings regarding the AI and aMCC were relatively unique to these individuals in comparison with those observed across all participants (Table 2).

No TMT index significantly explained EC with the WFU-defined pulvinar as seed. However, we found a significant negative relationship between response times on TMT-Part B and the strength of the ml-pulvinar seeded EC to the right amygdala (MNI: 27 −10 −20; 162 mm^3^; Z = 3.54, FWE-corrected *p* < 0.05 with small volume correction [SVC]) (Table S2), such that individuals with greater attentional control showed more enhanced EC from the ml-pulvinar to the amygdala (UF > UN). The PPI index extracted from this amygdala cluster had a marginally negative correlation with local amygdala activity (MNI: −21 −10 −17, Table 1) (*r* = −0.26, *p* = 0.099), suggesting that amygdala responses to UF (vs. UN) tended to be inhibited, possibly via the amygdala-pulvinar connection in individuals with greater attentional control.

## Discussion

Based on our initial hypothesis, we expected the pulvinar, along with the amygdala, to more respond to fearful faces emerging outside the focus of attention. However, unlike the amygdala, the pulvinar responses did not significantly differ between fearful faces and neutral faces in the background. These findings are consistent with the results of a previous study that showed similar increased activity in the amygdala, but not in the pulvinar, during the emotional face–house matching task in twelve subjects^8^. Although few studies have directly focused on the pulvinar’s role in emotional processing, a previous fMRI study examined the responses of the entire pulvinar to emotional stimuli and showed that fear-conditioned scene pictures presented among distractors in rapid serial presentation elevated pulvinar activity, but only in subjects who could capture these pictures^30^. This suggests that pulvinar responses to emotional stimuli may be influenced by specific factors, such as individual attentional ability or threat-sensitivity for capturing threatening stimuli among distractors.

In the present study, we examined the effects of threat-sensitivity (i.e., AB) and general attentional ability (i.e., TMT), and found a significant effect of AB, but not TMT, on local activity in the pulvinar. That is, individuals who quickly capture a threatening stimulus outside the focus of attention had significantly greater activity in the ml-pulvinar, which partially corresponds to the dorsomedial part (UF > UN). Additionally, individuals with greater AB had smaller grey matter volume in the medial pulvinar (Table S1, Figure S3). In line with these findings, physiological studies in animals have reported that pulvinar neurons (including its dorsomedial part) specifically respond to emotional entities, such as facial expressions or threatening animals, with firing onsets shorter than 50–100 ms^31^,^32^,^33^. Furthermore, in humans, the pulvinar damage is known to impair processing of visual threat, and particularly the damage of its medial portion is associated with incapability of recognizing fearful expressions^34^,^35^. These findings indicate that the pulvinar, at least some level of its dorsomedial part, may be relevant to emotion, especially to automatic attentional processing of threat, which depends on individual threat-sensitivity.

As predicted by our second hypothesis, we observed enhanced pulvinar-to-amygdala EC when fearful faces, as opposed to neutral faces, emerged outside the focus of attention. Although additional PPI analyses seeding in the amygdala specified that the pulvinar, predominantly its dorsomedial portion, was interconnected with the amygdala in a bilateral manner (Table S1), we observed significant EC from the left pulvinar (including whole subdivisions) to right amygdala. Structural connectivity between the amygdala and pulvinar is reported to be basically ipsilateral^12^. The contralateral synchronicity between the two regions however might occur via the SC, because a subset of fibers between the pulvinar and SC crossed the midline at the level of the intercollicular commissure and continued to the contralateral SC and pulvinar^12^. The discussion of the involvement of SC is still speculative and needs further investigation. The present results concur with those of an early seminal fMRI study that likewise reported elevated connectivity between these two regions when fear-conditioned angry faces were briefly presented without the participants’ consciousness^36^. These findings demonstrate that the pulvinar (particularly its dorsomedial parts) works with the amygdala, and possibly with the SC as well, during covert processing of emotional stimuli, although activation of the pulvinar itself is not significantly different between fearful and neutral stimuli. The pulvinar also showed greater EC to many other brain regions, including the attention-related FPN. A study combining diffusion tensor imaging and electrophysiological recordings in monkeys has revealed that the pulvinar causally regulates synchronized neural activity between cortical areas based on attentional allocation^37^. Given the pulvinar’s role in selective attention and its convergent and divergent projections to extensive brain areas^9^, as a key regulatory structure of selective attention, the pulvinar may drive not only the amygdala but also the attention-related FPN when it filters threats outside the focus of attention. This possibility partly conforms to the ‘multiple waves’ model of visual information, in which postulates that the pulvinar is an important node of extensive neural networks but not just a relaying structure to the amygdala^38^.

To further probe the meaning of the connectivity between the pulvinar and the amygdala, we tested whether AB could modulate the pulvinar-to-amygdala EC during the processing of UF (vs. UN). We expected an altered EC in individuals whose attention was biased toward threat. However, AB did not explain pulvinar-to-amygdala EC while TMT-B did. Specifically, the ml-pulvinar more strongly synchronized with the amygdala but not the FPN (Table S2), when fearful faces emerged from unattended locations in individuals with greater attentional control. Additionally, local amygdala activity became more attenuated in response to UF (vs. UN) as the synchronicity between these two regions increased. This result in part agrees with previous findings indicating that the magnitude of the amygdala’s response to unattended emotional stimuli was dependent on the levels of individual attentional resources^38^,^39^. Individuals with higher attentional control may have more resources left, allowing the pulvinar to inhibit emotional responses in the amygdala that may interfere with focused attention to a target stimulus.

Threat-sensitive individuals (i.e., greater AB) exhibited enhanced EC from the ml-pulvinar to the OP, the precentral gyrus, SMG, SMA, aMCC, and AI, when fearful faces (as opposed to neutral faces) emerged from unattended locations. These FPN regions constitute two main networks: the dorsal network (e.g., the SPL and SMA) responsible for endogenous attention, and the ventral network (e.g., the SMG, OP, and AI) responsible for exogenous attention (the aMCC has been shown to be connected to both networks)^3^,^4^. While the connectivity patterns in threat-sensitive individuals were similar to those observed across all participants, the EC to the ventral network regions and the aMCC was relatively unique in these individuals. The aMCC, together with the OP and AI, is known to form a neural network, called the midcingulo-insular-inferior frontal (MCC-IS-IF) network, which plays a supervisory role in attentional control, as shown by a recent neuroimaging meta-analytic study^40^. Individuals with greater AB tend to have poorer attentional ability, as seen in this study (Table S3) and in our previous study, which used a sizable sample^41^. They may thus have a strengthened EC from the pulvinar to MCC-IS-IF regions, which may serve to augment attentional control and maintain focused attention on a target stimulus when fearful faces appear in a distracting manner. Therefore, the pulvinar may decide which region is more strongly driven so that it can maintain selective attention on a goal stimulus when it filters threats outside the focus of attention whose connectivity patterns are dependent on individual differences in threat-related AB and attentional control.

There are several limitations that should be noted. First, behavioural data, including eye movement during fMRI, were not measured in this study. The issue was limited, however, since the previous studies consistently confirms rare saccades and no major difference in eye position associated with experimental factors during the face-house matching task^8^,^42^,^43^,^44^, in addition to no significant effect on activity in the pulvinar during eye movement^9^. All participants reported that they were unable to recognize the facial expressions of background faces during the scan. Second, we used 1.5-T MRI, whose relatively lower spatial resolution may impact the detection of significant differences in small brain structures. However, 1.5-T MRIs are still useful in detecting signal changes in deeply-embedded structures such as the amygdala. Additionally, we used the entire pulvinar as the ROI in this study, because of the low resolution. This limits the precise differentiation of BOLD activities between the pulvinar and SC because the two regions are spatially close to each other. Lastly, causal relationships between the pulvinar and its connected regions were unclear. Future studies leveraging a higher-resolution functional device (e.g., 7T-fMRI) and other neural recording devices such as electroencephalography and magnetoencephalography, which overcome the low temporal and spatial resolution of the existing fMRI, will further delineate the exact functions of the human pulvinar, differentiating its subdivisions and interconnections.

In conclusion, the pulvinar responses did not significantly differ between fearful faces and neutral faces at unattended locations, whereas the amygdala did. However, the pulvinar (particularly its dorsomedial part) strongly synchronized with the amygdala, suggesting that these two regions work closely during processing of fearful faces that covertly appeared in the background. Notably, individuals who promptly direct selective attention to threat (i.e., AB) showed significantly increased activity (UF > UN) in the pulvinar (including a dorsomedial part), as well as its reduced volume. These individuals further exhibited more strengthened EC from the pulvinar to the FPN, but not the amygdala, during the processing of UF (vs. UN). In contrast, individuals with greater attentional control showed strengthened EC from the pulvinar to the amygdala, but not the FPN. The pulvinar, or at least some level of dorsomedial pulvinar, may filter emotional stimuli outside the focus of attention whose sensitivities depend on individual differences in threat-related attentional bias. Further, pulvinar connectivity patterns during the processing of unattended emotional stimuli may be determined based on individual attentional control and attentional bias toward threat.

## Methods

### Participants

The Kitasato University Hospital Institutional Review Board approved the study. All experimental methods were carried out in accordance with the ethical guidelines determined by the National Ministry of Health, Labour and Welfare and the Declaration of Helsinki. All participants provided written informed consent. The participants were recruited via advertisements in a local magazine and on billboards at Kitasato University. The eligibility criteria were as follows: no Axis-I psychiatric disorders or substance-abuse history as determined by the Mini-International Neuropsychiatric Interview^45^; no major medical/neurological illnesses; no head injury with loss of consciousness; no history of habitual smoking; and no left-handedness as assessed by the Edinburgh Handedness Inventory^46^. Six subjects were excluded due to head movement (*n* = 2) and MRI artefact (*n* = 4). Eventually, forty-one healthy right-handed subjects participated in the present study (25 women; mean age: 21.9 years, range: 20–33, standard deviation [SD] = 2.9).

### Psychological Assessment

We used the TMT^47^,^48^ to assess attentional control. TMT is one of the most representative neuropsychological tests for the assessment of attentional function and consists of two parts: TMT-A (which requires participants to connect randomly distributed numbers consecutively with a line on paper) and TMT-B (in which numbers and letters are connected in an alternating fashion). Response time (RT) (measured in seconds) indexes focused and divided attention in TMT-A and TMT-B, respectively. TMT-A specifically assesses the ability to focus on a specific stimulus, while TMT-B tests the ability to divide attention smoothly between two different cognitive categories, and is thereby specifically associated with attentional control^49^. Longer RTs indicate lower attentional ability.

To assess AB, we used the dot-probe task (DPT)^50^, which is the most widely used test for this purpose (Figure S1). Details of the DPT are described elsewhere^47^. Briefly, a pair of words differing in emotional valence (threat or non-threat) are presented at the same time for 500 ms, and are then immediately followed by a probe (i.e. asterisk) appearing at one of the two words’ locations. The probes are presented with equal frequencies (i.e. 50% appearing at each location). The participants were asked to press one of two buttons as quickly and accurately as possible to indicate the location of the probe. The location of the probe was counterbalanced across 196 trials. The presentation order of the paired words was randomized for each participant. Differences between RT for neutral stimuli and RT for negative stimuli served as an index of AB. Positive values indicate bias toward threat.

### fMRI experimental design

**Figure 6:**
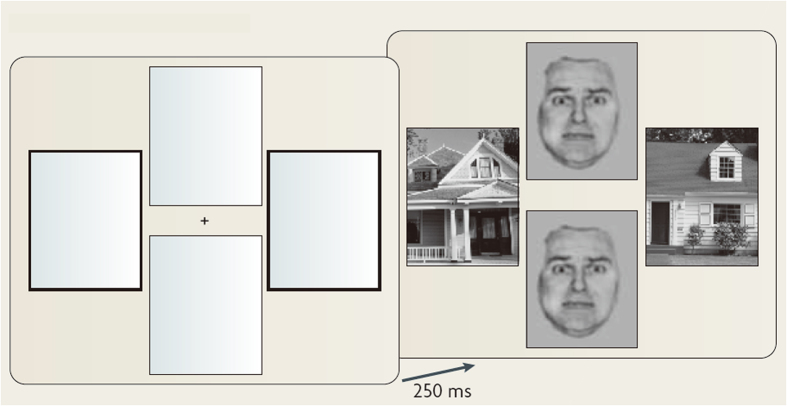
Experimental paradigm. The first display presents four empty frames in the vertically and horizontally paired positions, with either the two horizontal or the two vertical frames being highlighted. The next display shows a central fixation point followed by paired faces and paired houses arranged in vertical or horizontal positions. The two highlighted frames always predict the locations of the paired houses in the present study. Participants were required to attend to the paired houses preceded by the two highlighted frames and to judge whether the houses were the same or different. Both faces had either neutral or fearful expressions. The positions of the paired faces or houses (vertical or horizontal) and the facial expressions (neutral or fearful) were counterbalanced across subjects and were presented in a random order. The figure is reprinted with permission from the ref. 5 © Macmillan Publishers Ltd., in Nature Publishing Group. The figure is a modified version of the original figure from the ref. 8 © Cell Press.

A dual-task design was employed using a modified version of the face-house matching task^8^,^42^. All stimuli consisted of displays of four pictures, with two faces and two houses arranged in vertical and horizontal pairs around a fixation point (Fig. 6).

All pictures were black and white photographs (visual angle 18° × 13°) presented on a black background and projected through a mirror mounted onto the head coil. The schedule of stimulus presentation was controlled by the E-prime 2.0 software. For face stimuli, eight different Japanese faces with fearful and neutral expressions (four women and four men) were selected from the ATR facial database (DB99; ATR-Promotions, Inc., Kyoto, Japan). We selected eight houses to serve as the house stimuli. In each trial, after a 2-second visual display consisting of four empty frames in the vertically- or horizontally-paired positions with either the two horizontal or the two vertical frames being highlighted, a fixation point was presented at the centre of the screen for 1 second. This was followed by the presentation of paired faces and paired houses at the locations of the frames for 250 ms. The stimulus intervals varied randomly between 3.5 and 12.5 seconds for the 48 trials, with a mean of 7.5 seconds. Participants were instructed to attend to the paired houses preceded by the two highlighted frames and to judge whether or not the houses are identical. The positions of the paired faces or houses (vertical or horizontal) and the types of facial expressions (neutral or fearful) were presented in a random order for each subject. Both faces in each pair had the same facial expression on a given trial. To help us focus on pulvinar activity to unattended fearful faces, the paired houses were always preceded by two highlighted frames. This led to the presentation of the two conditions (UF and UN).

### MRI scanning

Anatomical and functional MRI scans were acquired using a 1.5-T GE Signa scanner (GE Medical Systems, Milwaukee, Wisconsin) with an eight-channel phased-array head coil. Structural images were acquired with a 3D T1-weighted sequence (1.2-mm slice thickness without space; field of view (FOV) = 240 mm; matrix = 288 × 256; repetition time (TR) = 13.5 ms; echo time (TE) = 5.8 ms; and flip angle (FA) = 20°). For functional images, data were acquired using fast-gradient-echo-planar T2*-weighted imaging with five dummy volumes at the beginning of the session. Each functional volume consisted of 30–34 transverse slices (4.0-mm thickness and 1.0-mm slice gap; FOV = 260 mm; matrix = 128 × 128; TR = 3,000 ms; TE = 40 ms; FA = 90°).

### fMRI data analysis

#### Comparison between UF vs. UN

Data were analysed using a general linear model (GLM) for this event-related design in Statistical Parametric Mapping software (SPM8; Institute of Neurology, University College London, London, UK)^51^,^52^,^53^. Pre-processing procedures are provided in the Supplementary Material. After pre-processing, we used GLM to perform 1st-level analysis for each participant to obtain parameter estimates of event-related activity at each voxel for each condition (UF or UN). We also created individual contrast maps consisting of voxels of linear contrasts between the conditions. These individual contrast maps were used in 2nd-level random-effects analysis to identify statistically significant voxels across participants between the two conditions (UF > UN or UF < UN).

#### PPI analysis

We conducted PPI analysis to detect voxels showing task-specific increases between a seed region of interest and the rest of the brain^54^,^55^. Since we expected to observe significant pulvinar-to-amygdala EC, we selected the pulvinar as a seed region (Figure S2). Three regressors were included in the analysis: (1) the contrast between the two conditions (the psychological regressor), (2) the time course in the pulvinar (the physiological regressor), and (3) the interaction term for these two regressors (the PPI regressor). Based on our hypotheses, we focused on the contrast of UF minus UN. The first regressor represented the main effect of task (UF > UN), which was convolved with the hemodynamic response function (HRF). The second regressor was the blood oxygenation level dependent (BOLD) time course of the seed region, which was extracted in terms of the first eigenvariate from a 5-mm radius sphere around each individual’s maximum activation (UF > UN) in the pulvinar, as defined by the WFU PickAtlas software^56^,^57^. This regressor was extracted separately for the right and left sides of the pulvinar. The last PPI regressor was created as follows: (1) the pulvinar seed BOLD signals were deconvolved with HRF and transformed into the neural level; (2) the element-by-element product of the neural-level pulvinar time series and the psychological contrast was calculated; (3) the interactive product was reconvolved with the HRF and transformed again onto the hemodynamic level to explore the hemodynamic signals for each voxel. These three regressors were entered into a GLM, which was estimated for each participant. The resulting individual contrast images, which were estimated from the last PPI term (UF > UN), were entered into the 2nd-level between-subject analysis, with age and sex as covariates.

We also performed another PPI analysis by placing a seed region on a portion of the ml-pulvinar whose activity was significantly predicted by AB (UF > UN) (Fig. 4, peak MNI coordinate: 24 −31 1). We used a smaller region for our analyses, since the pulvinar, as defined by the WFU PickAtlas, may be too large to identify significant EC to the amygdala due to its anatomical complexity. Within the cluster, the second regressor BOLD time course was extracted from the 5-mm radius sphere around each individual’s maximum activation (UF > UN). The other regressors were calculated with the same procedure as the one used for PPI analysis of the WFU pulvinar seeds.

#### Effect of emotional and non-emotional attention on pulvinar activity and its EC

We explored a possible modulatory effect of AB on the activity of the pulvinar and its EC to the amygdala. We conducted separate whole-brain regression analyses using SPM, with AB as an explanatory variable, and age and sex as nuisance variables for the individual contrasted images showing local activity (UF > UN) and for images showing connectivity (PPI). To ensure that a pulvinar cluster is specifically relevant to emotional attention but not to non-emotional attention, similar analyses were applied to TMT indices. Detailed procedures are described in the Supplementary Material.

### Statistical thresholding

Results were presented at a cluster-size threshold of *p* = 0.05, corrected for multiple comparisons by FWE. Because of our main interest in the pulvinar and the amygdala, we adopted the region of interest (ROI) approach and set the ROIs as the pulvinar and amygdala. These regions were defined by anatomical masks in the WFU PickAtlas. Although our main interest was in the relationship between the pulvinar and amygdala, we also included the midbrain (WFU PickAtlas-defined) as well in the ROIs, considering direct visual inputs from the SC to pulvinar. We used SVC for the clusters found within these ROIs in each analysis (*p* < 0.001 with extent threshold *k* > 5). We adopted a liberal threshold for the exploratory analysis of the results of the regression analysis for the PPI contrasts using the right ml-pulvinar as a seed (uncorrected *p* < 0.001 with *k* > 5). The procedures used to identify the pulvinar subnuclei are provided in the Supplementary Material and Figure S2.

## Additional Information

**How to cite this article**: Hakamata, Y. *et al*. The functional activity and effective connectivity of pulvinar are modulated by individual differences in threat-related attentional bias. *Sci. Rep.* **6**, 34777; doi: 10.1038/srep34777 (2016).

## Supplementary Material

Supplementary Information

## Figures and Tables

**Table 1:** Brain regions demonstrating significantly increased or decreased activity in response to UF vs. UN.

Side	Cortical regions	volume (mm^3^)	*Z*	Coordinates		
				x	y	z
UF > UN†						
L	amygdala*	351	3.94	−21	−10	−17
R	amygdala	189	3.76	21	−10	−17
UF < UN						
No brain region was found to be significant.						

UF, unattended fearful faces condition; UN, unattended neutral faces condition; L, left; R, right; SVC, small volume correction.

Regions were significant at *p* < 0.001 with SVC (regions of interest: the pulvinar and amygdala).

^*^The cluster was significant at FWE-corrected *p* < 0.05 with SVC.

^†^When the midbrain was included in the ROIs, the left amygdala cluster extended to the ventral midbrain, including a part of parahippocampal region (peak MNI: −9 −10 −11, 216 mm^3^; Z = 3.83, uncorrected *p* < 0.001).

**Table 2:** Brain regions showing significantly stronger synchrony with the pulvinar in response to UF vs. UN in PPI analyses.

Side	Cortical regions	volume (mm^3^)	*Z*	Coordinates		
				x	y	z
Seed: left pulvinar (defined by WFU PickAtlas)†						
FWE-corrected *p* > 0.05						
R	fusiform gyrus	270	5.54	33	−52	−17
R	cuneus	405	5.25	9	−76	10
L	operculum, inferior frontal gyrus	108	5.10	−54	11	31
R	fusiform gyrus	27	4.81	42	−43	−23
R	inferior temporal gyrus	27	4.81	45	−52	−14
*p* > 0.001 with SVC						
R	amygdala*	135	3.57	30	−1	−26
Seed: right pulvinar (defined by WFU PickAtlas)§						
FWE-corrected *p* > 0.05						
R	supplementary motor area	81	5.28	3	23	49
L	middle occipital gyrus	54	5.25	−39	−82	16
L	precentral gyrus	54	5.15	−36	−25	58
R	superior parietal lobule	27	4.99	27	−58	43
R	middle occipital gyrus	27	4.89	39	−73	19
L	midcingulate gyrus	27	4.82	−6	14	40
L	declive, cerebellum	27	4.81	−36	−61	−26
L	inferior occipital gyrus	54	4.79	−45	−73	−11
Seed: a part of the right ml-pulvinar (AB-related cluster)						
FWE-corrected *p* > 0.05						
R	supplementary motor area	108	5.18	54	−28	46
R	supramarginal gyrus	81	5.10	3	−1	52

PPI, psychophysiological interaction; ml-pulvinar, a part of the medial and lateral pulvinar nuclei; L, left; R, right; UF, unattended fearful faces condition; UN, unattended neutral faces condition. SVC, small volume correction; AB, attentional bias.

SVC was applied to the region of interest (i.e., the amygdala).

^*^The cluster was significant at FWE-corrected *p* < 0.05 with SVC.

^†^The cluster adjacent to the SC was observed in the dorsal midbrain (peak MNI: 15 −16 −2, 675 mm^3^; Z = 3.92, uncorrected *p* < 0.001), when the midbrain was considered.

^§^The cluster was likewise observed in the dorsal midbrain, which was close to the reticular formation (peak MNI: 15 −25 −11, 297 mm^3^; Z = 4.16, uncorrected *p* < 0.001).

**Table 3:** Brain regional activity significantly correlated with attentional bias toward threat: UF > UN.

Side	Cortical regions	Volume (mm^3^)	*Z*	Coordinates		
				x	y	z
Positive correlation						
R	pulvinar (medial and lateral parts)*	135	3.54	24	−31	1
Negative correlation						
No brain region was found to be significant.						

L, left; R, right; SVC, small volume correction; UF, unattended fearful faces condition; UN, unattended neutral faces condition.

Pulvinar nuclei defined by Morel’s 3D atlas of the human thalamus. Anterior, medial, inferior, and lateral nuclei are abbreviated as PuA, PuM, PuI, and PuL, respectively.

The region was significant at *p* < 0.001 with SVC (regions of interest: the pulvinar and amygdala).

No significant correlation was found for UF < UN.

^*^Overlapped with Morel’s pulvinar subnuclei: PuL: *x* = 24, −34 ≤ *y* ≤ −28, −2 ≤ z ≤ 2, 48 mm^3^; PuM: 20 ≤ *x* ≤ 24, −34 ≤ *y* ≤ −28, −2 ≤ z ≤ 0, 128 mm^3^.

**Table 4:** The results of PPI analysis using the right ml-pulvinar as a seed: correlation with attentional bias.

Side	Cortical regions	volume (mm^3^)	*Z*	Coordinates		
				x	y	z
Positive correlation with AB						
R	operculum, inferior frontal gyrus	162	4.09	48	14	7
R	precentral gyrus	297	4.08	33	−4	37
R	precuneus	540	4.07	24	−46	46
R	supramarginal gyrus	270	3.90	63	−25	19
L	cingulate gyrus, including portions of the medial superior frontal gyrus and supplementary motor area	513	3.90	−6	17	40
			3.53	−12	11	37
R	midcingulate gyrus	135	3.59	6	8	40
L	anterior insula	162	3.54	−36	14	7
L	supplementary motor area	189	3.46	−9	8	49
R	midcingulate gyrus	216	3.35	6	−13	37
Negative correlation with AB						
No brain region was found to be significant.						

PPI, psychophysiological interaction; ml-pulvinar, a part of the medial and lateral pulvinar nuclei; L, left; R, right; UF, unattended fearful faces condition; UN, unattended neutral faces condition.

The ml-pulvinar, the seed region, was determined based on the cluster that was significantly correlated with attentional bias (MNI coordinate: 24 −31 1).

The table shows the brain regions that were significant at a liberal threshold, uncorrected *p* < 0.001 and *k* < 5 for the exploratory analysis.

Results were based on the contrast of UF > UN.
