# Supplementary material for: The functional activity and effective connectivity of pulvinar are modulated by individual differences in threat-related attentional bias
Source: Sci Rep. 2016 Oct 5;6:34777. doi: 10.1038/srep34777 (PMC5050502; doi:10.1038/srep34777)
Supplement: Supplementary Information [file srep34777-s1.pdf]

The functional activity and effective connectivity of pulvinar are modulated by individual differences in threat-related attentional bias

Authors: Yuko Hakamata[1,2,3*], Eisuke Sato[4], Shotaro Komi[5], Yoshiya Moriguchi[6], Shuhei Izawa[7], Norio Murayama[2], Takashi Hanakawa[6], Yusuke Inoue[8], Hirokuni Tagaya[2]

Affiliations: [1]Department of Adult Mental Health, National Institute of Mental Health, National Center of Neurology and Psychiatry; [2]Department of Health Sciences, Kitasato University School of Allied Health Sciences; [3]Department of Clinical Psychology, Graduate School of Education, The University of Tokyo; [4]Department of Medical Radiological Technology, Kyorin University School of Health Sciences; [5]Department of Clinical Engineering, Kitasato University School of Allied Health Sciences; [6]Integrative Brain Imaging Center, National Center of Neurology and Psychiatry; [7]Department of Health Administration and Psychosocial Factor Research Group, National Institute of Occupational Safety and Health; [8]Department of Diagnostic Radiology, Kitasato University School of Medicine

***Corresponding author:**

Yuko Hakamata, Ph.D.

Department of Adult Mental Health,
National Institute of Mental Health, National Center of Neurology and Psychiatry,
4-1-1, Ogawahigashi, Kodaira, Tokyo, 187-8553, Japan.
Phone: +81 42 341 2711; fax: +81 42 346 1986.

Email: yumhakamata@gmail.com

Supplementary Material

1. METHODS

1.1. Functional magnetic resonance imaging (fMRI) pre-processing

Prior to statistical analysis, the following steps were applied to the scanning data: realignment, slice-time correction, spatial normalization to the Montreal Neurological Institute (MNI) space, and Gaussian spatial smoothing (full width at half maximum [FWHM]: 6 mm) [1].

1.2. Structural MRI (sMRI) pre-processing

Voxel-based morphometry (VBM) [2, 3] was performed using SPM8. Pre-processing procedures were as follows: realignment; segmentation of images into probability maps of grey matter (GM), white matter (WM), and cerebrospinal fluid (CSF); creation of a population GM template; spatial normalization of the population template to the MNI space; and Gaussian spatial smoothing (FWHM: 10 mm). Prior to the spatial normalization, GM maps of each subject were nonlinearly warped to the population GM template and then transformed according to the Jacobian determinant map using DARTEL, which enables accurate inter-subject registration of brain images [4].

1.3. fMRI data analysis

1.31. Effect of non-emotional attention on pulvinar activity and its effective connectivity (EC)

Whole-brain analysis

We conducted whole-brain regression analyses with Trail Making Test (TMT) indices as predictors for individual contrast images (UF > UN) and for individual psychophysiological interaction (PPI) contrast images (UF > UN), respectively. We performed these analyses to confirm that the pulvinar has a specific role in emotional, but not non-emotional, attention.

Region of interest (ROI)-based analysis

ROI-based analyses were performed to ensure that the clusters observed in the pulvinar were solely explained by individual differences in emotional attention (i.e., attentional bias [AB]), but not those in non-emotional general attention (i.e., TMT). For the clusters in the pulvinar that were found to be significantly correlated with AB, estimated values (βs) were extracted with MarsBar 0.43 (http://marsbar.sourceforge.net/) [5]. With each β value as a dependent variable, TMT indices (TMT-A and TMT-B) were incorporated into each step-wise regression model as possible predictors. These ROI-based regression analyses were performed with SPSS 19.0J (IBM, Inc., Tokyo, Japan). The statistical significance threshold was set at $p < 0.05$ (two-tailed). Age and sex were controlled for in the analyses.

1.32. Additional psychophysiological (PPI) analysis seeding in the amygdala

We further performed PPI analyses seeding in the amygdala in order to specify which subdivision of the pulvinar was connected with the amygdala. The amygdala was defined based on the WFU PickAtlas in the left and right side separately. The procedures were the same as the other PPI analyses. Specification of pulvinar subdivisions was performed according to the following

procedures ("**1.5. Identification of pulvinar subnuclei**"). We reported the results that small volume correction (SVC) was applied to, using the WFU pulvinar mask as the ROI , at $p < 0.001$ without extent threshold, due to possible small volume of a subdivision of the pulvinar (e.g., inferior pulvinar).

1.4. sMRI data analysis: correlation of GM volume with AB and with TMT

We performed three separate regression analyses on the VBM images with AB, TMT-A, and TMT-B, as independent variables, controlling for age, sex, and intracranial volume.

1.5. Identification of pulvinar subnuclei

We used the unbiased, high-resolution, 3D atlas of the human thalamus, which was anatomically defined by Morel [6, 7], to determine which subdivision of the pulvinar each observed cluster was located. This model highly corresponds to the model obtained by the meta-analytic connectivity modelling and coactivation-based parcellation in a large number of human neuroimaging studies [8]. In Morel's atlas, the pulvinar has four subdivisions: anterior, medial, inferior, and lateral nuclei (PuA, PuM, PuI, and PuL, respectively) (Figure S3). The location of each cluster observed in the pulvinar was anatomically compared to each subnucleus of Morel's pulvinar using the 'combine ROIs' option in MarsBar, which calculates an overlapped region between the observed cluster and each subnucleus.

2. RESULTS

2.1. fMRI

2.11. Effect of non-emotional attention on pulvinar activity and its EC

Whole brain analysis

No TMT index had any correlation with functional activity in the whole brain.

ROI-based analysis

We observed that the right medial and lateral pulvinar nuclei (ml-pulvinar) significantly correlated with AB (Table 3, Figure 4). After extracting the blood oxygen level dependent (BOLD) signals from the cluster found in the preceding analysis (MNI coordinate: 24 −31 1, 135 mm^3), we performed a step-wise multiple regression analysis with TMT indices as candidate independent variables. As a result, TMT-A was observed to be a significant *positive* predictor of ml-pulvinar activity (UF > UN) ($F(1,39) = 5.071$, $R^2 = 0.12$, adjusted $R^2 = 0.09$, standardized $\beta = 0.34$, $p = 0.03$), suggesting that the ml-pulvinar was more strongly activated by UF (vs. UN) in individuals who had more difficulty maintaining focused attention on a target stimulus. Nevertheless, when AB was included in the step-wise regression model as an independent variable, AB was selected solely as a dominant explanatory variable and TMT-A was no longer a significant predictor (AB as a significant predictor: $F(1,39) = 16.49$, $R^2 = 0.30$, adjusted $R^2 = 0.28$, standardized $\beta = 0.55$, $p < 0.001$; TMT-A as an excluded variable: standardized $\beta = 0.09$, $p = 0.55$). The result suggests that activity of the ml-

pulvinar was affected by individual dispositions in emotional attention, not by differences in non-emotional general attention.

2.12. PPI analyses seeding in the amygdala

Results were presented in Table S1. We found significantly increased synchronicity between the amygdala and three pulvinar subdivisions bilaterally: the medial, anterior, and lateral parts (Table S1). Particularly, the right amygdala showed significantly enhanced connectivity with the right mediolateral pulvinar, the largest cluster, during the processing UF compared to UN (Figure S4).

2.2. sMRI

We found that greater AB significantly predicted a smaller GM volume in the bilateral medial pulvinar (Table S2, Figure S4). In contrast, no TMT indices predicted any structural volume changes. To ensure that the clusters observed in the medial pulvinar (Table S2, Figure S4) were solely explained by individual differences in AB (emotional attention), but not TMT (non-emotional attention), we performed a step-wise multiple regression analysis with TMT measures as potential explanatory variables for the β-values extracted from each cluster in the bilateral medial pulvinar. However, none of these TMT variables was found to be a significant predictor of GM volume in the medial pulvinar, corroborating the specific relevance of the pulvinar to emotional attention.

3. DISCUSSION

We observed a significant effect of AB on functional activity as well as structural volume: individuals with greater AB showed significantly greater ml-pulvinar activity in response to UF (vs. UN) and had a smaller GM volume in the medial pulvinar. These relationships were not predicted by TMT indices. It is difficult to give an integrative account for the greater activity and smaller pulvinar GM volume observed in individuals with biased attention toward threat. Reduced GM volume in the amygdala has been suggested to mediate the exaggerated activity to emotional stimuli in patients with post-traumatic stress disorder [9-13]. Similarly, the smaller GM volume of the pulvinar may lead to its greater activity in these threat-sensitive individuals, although this interpretation will clearly need further scrutiny.

Supplementary tables

Table S1. Subnuclei of the pulvinar showing significantly increased synchronicity with the amygdala in PPI analyses (UF > UN)

	Side	Subnucleus	Overlap with Morel's defined pulvinar (mm^3)	volume (mm^3)	*Z*	Coordinates		
						x	y	z
**Seed: left amygdala (WFU PickAtlas defined)**								
*	L	lateral and medial parts	PuL ($32mm^3$), PuM ($8mm^3$)	108	3.52	-24	-31	4
*	L	medial and anterior parts	PuM ($48mm^3$), PuA ($16mm^3$)	81	3.27	-12	-25	7
*	R		*Not specified*	54	3.27	15	-25	13
*	L		*Not specified*	27	3.19	-21	-25	16
**Seed: right amygdala (WFU PickAtlas defined)**								
*	R	medial, lateral, and anterior parts	PuM ($120mm^3$), PuL ($64mm^3$), PuA ($8mm^3$)	405	3.75	24	-25	7
*	L	medial part	PuM ($32mm^3$)	108	3.48	-15	-34	1
*	R		*Not specified*	27	3.20	9	-28	1

PPI, psychophysiological interaction; UF, unattended fearful faces condition; UN, unattended neutral faces condition; L, left; R, right; SVC, small volume correction.

SVC was applied to the region of interest (i.e., the pulvinar) at $p < 0.001$ without extent threshold.

*The cluster was significant at FWE-corrected $p < 0.05$ with SVC.

Pulvinar nuclei defined by Morel's 3D atlas of the human thalamus. Anterior, medial, inferior, and lateral nuclei are abbreviated as PuA, PuM, PuI, and PuL, respectively.

Table S2. Brain regional volumes significantly correlated with AB

Side	Cortical regions	volume (mm^3)	*Z*	Coordinates		
				x	y	z
Positive correlation with AB						
No brain region was found to be significant.						
Negative correlation with AB						
R	mediodorsal nucleus	138	3.41	5	-20	5
	medial pulvinar[*1]		3.34	12	-27	5
L	mediodorsal nucleus	23	3.34	-5	-20	6
L	medial pulvinar[*2]	20	3.20	-9	-27	5

L, left; R, right; SVC, small volume correction; AB, attentional bias.

Regions were significant at uncorrected $p < 0.001$ with SVC (ROIs: the pulvinar and the amygdala).

Pulvinar subnuclei defined by Morel's 3D atlas of the human thalamus; anterior, medial, inferior, and lateral nuclei are abbreviated as PuA, PuM, PuI, and PuL, respectively.

∗1 overlapped with PuM: $-12 \leq x \leq 14$, $-28 \leq y \leq -26$, $4 \leq z \leq 6$, 24 mm^3.

*2 overlapped with PuM: x = -10, y = -28, z = 4, 8 mm^3.

Table S3. Results of PPI using the right ml-pulvinar as a seed: correlation with TMT-B

Side	Cortical regions	volume (mm^3)	*Z*	Coordinates		
				X	y	z
**Positive correlation with TMT-B**						
No brain region was found to be significant.						
**Negative correlation with TMT-B**						
R	putamen, including the hippocampus and amygdala*	1350	4.65	27	-16	-8
			3.54	27	-10	-20
R	postcentral gyrus	162	3.93	39	-28	46

PPI, psychophysiological interaction; ml-pulvinar, a part of the medial and lateral pulvinar nuclei; TMT-B, Trail Making Test-Part B; L, left; R, right; UF, unattended fearful faces condition; UN, unattended neutral faces condition.

The ml-pulvinar, the seed region, was determined based on the cluster that was significantly correlated with attentional bias (MNI coordinate: 24 -31 1)

The table shows brain regions that were significant at a liberal threshold, as in the PPI results on AB in Table S1 (i.e., uncorrected $p < 0.001$ and $k < 5$). Results were based on the contrast of UF > UN.

*The cluster (MNI coordinate: 27 -10 -20; 162 mm^3; Z = 3.54) survived a cluster-sized FWE-corrected $p = 0.05$ with small volume correction (ROI: the amygdala).

TMT-A did not predict any relationship in PPI analysis.

Table S4. Correlation between attentional bias toward threat and TMT indices

	Attentional bias score	TMT-A RT (s)	TMT-B RT (s)
Attentional bias score	1		
TMT-A RT (s)	0.49	1	
	0.001		
TMT-B RT (s)	0.28	0.40	1
	0.07	0.01	

TMT, Trail Making Test. Values in upper and lower rows indicate correlation coefficients and *p*-values, respectively.

Supplementary figures

Figure S1

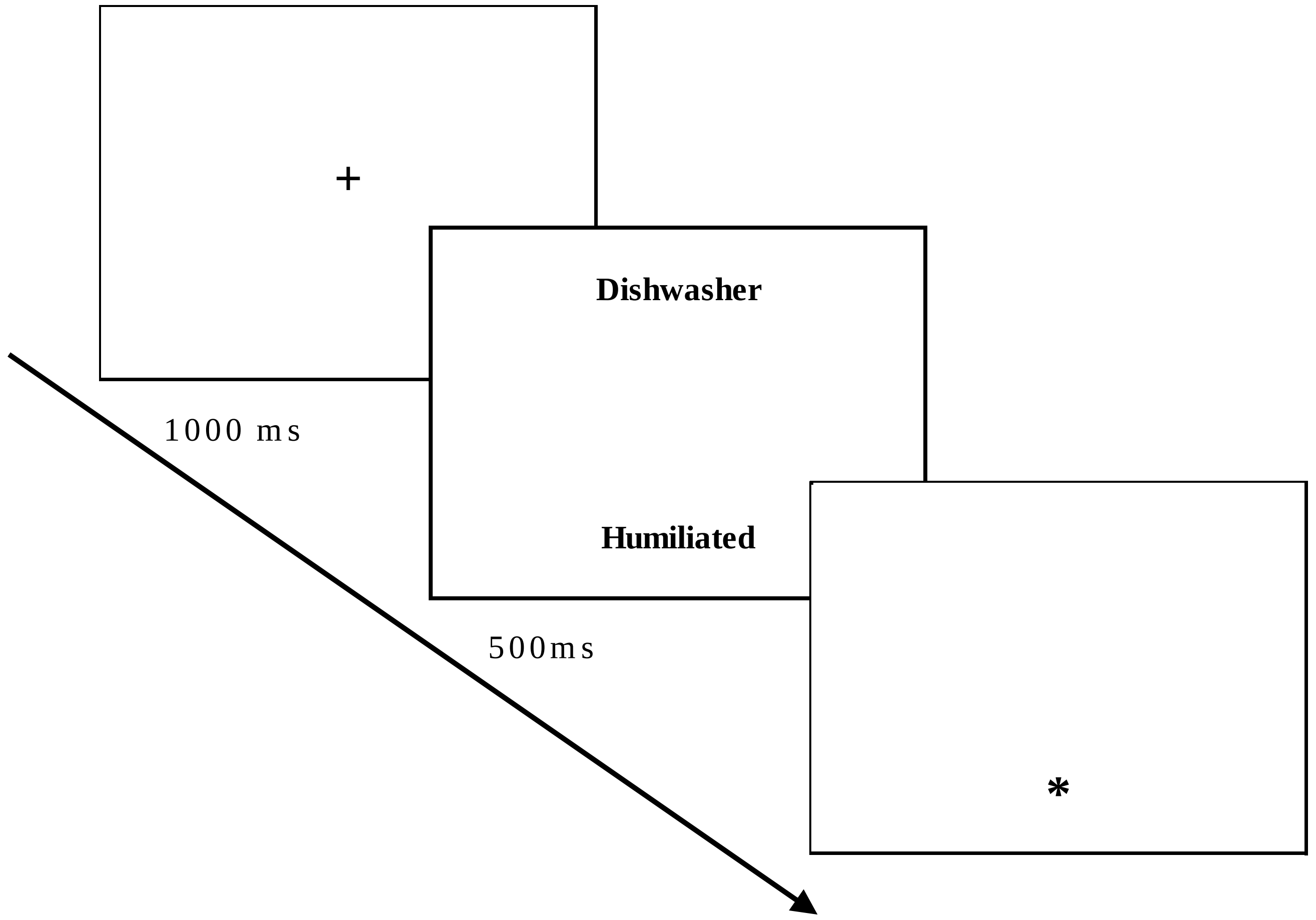

Figure S1. Dot-probe task. A pair of words differing in emotional valence (threat or non-threat) are presented at the same time for 500 ms and then immediately followed by a probe (i.e. asterisk) appearing at one of the locations where the two words were presented with equal frequency (i.e. 50% at each position).

Figure S2

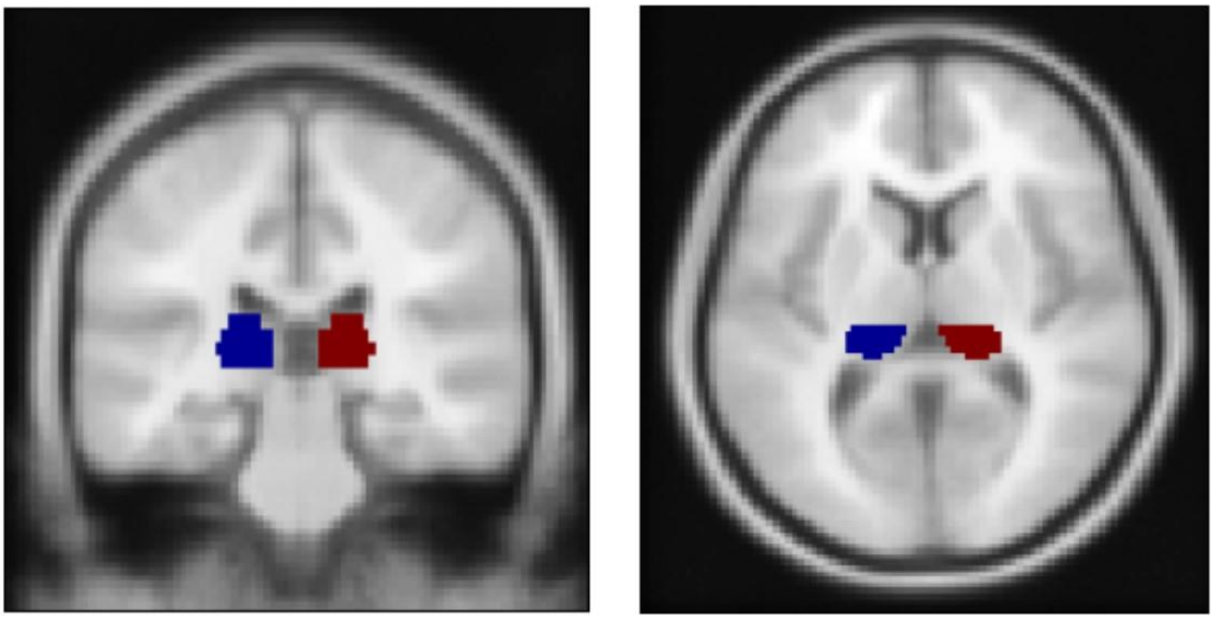

Figure S2. Pulvinar as a seed region in psychophysiological interaction analyses.

Seed region was defined based on the pulvinar of the WFU PickAtlas. Left pulvinar (blue colour). Right pulvinar (red colour). The coronal plane image is presented at *y* = -28. The transverse plane image is presented at *z* = 8.

Figure S3.

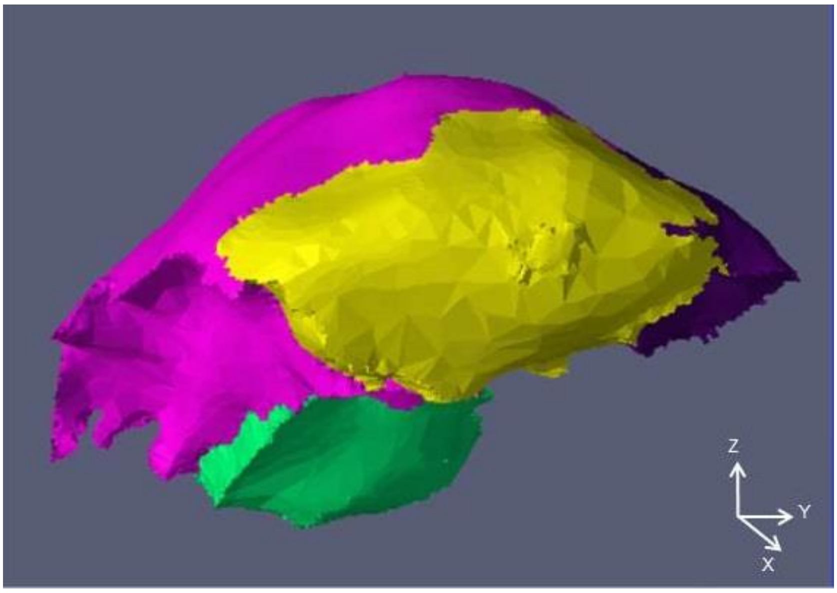

Figure S3. Pulvinar nuclei defined by the 3D atlas of the human thalamus [15]. The 3D atlas was invented based on anatomical definitions by Morel et al. [7, 8].

Pink colour = medial pulvinar nucleus (PuM); yellow colour = lateral pulvinar nucleus (PuL); purple colour = inferior pulvinar nucleus (PuI); green colour = anterior pulvinar nucleus (PuA). Right side: PuM: $6 \le x \le -24$, $-36 \le y \le -24$, $-5 \le z \le 14$, 1833 mm^3; PuL: $21 \le x \le 28$, $-34 \le y \le -25$, $-3 \le z \le 11$, 397 mm^3; PuI: $22 \le x \le 27$, $-32 \le y \le -27$, $-6 \le z \le -2$, 62 mm^3; PuA: $12 \le x \le 18$, $-26 \le y \le -21$, $-1 \le z \le 9$, 169 mm^3. Left side: PuM: $-23 \le x \le -4$, $-36 \le y \le -23$, $-4 \le z \le 14$, 1832 mm^3; PuL: $-26 \le x \le 20$, $-33 \le y \le -25$, $-3 \le z \le 11$, 388 mm^3; PuI: $-25 \le x \le -20$, $-32 \le y \le -27$, $-6 \le z \le -2$, 59 mm^3; PuA: $-17 \le x \le -10$, $-26 \le y \le -21$, $-1 \le z \le 9$, 167 mm^3.

Figure S4

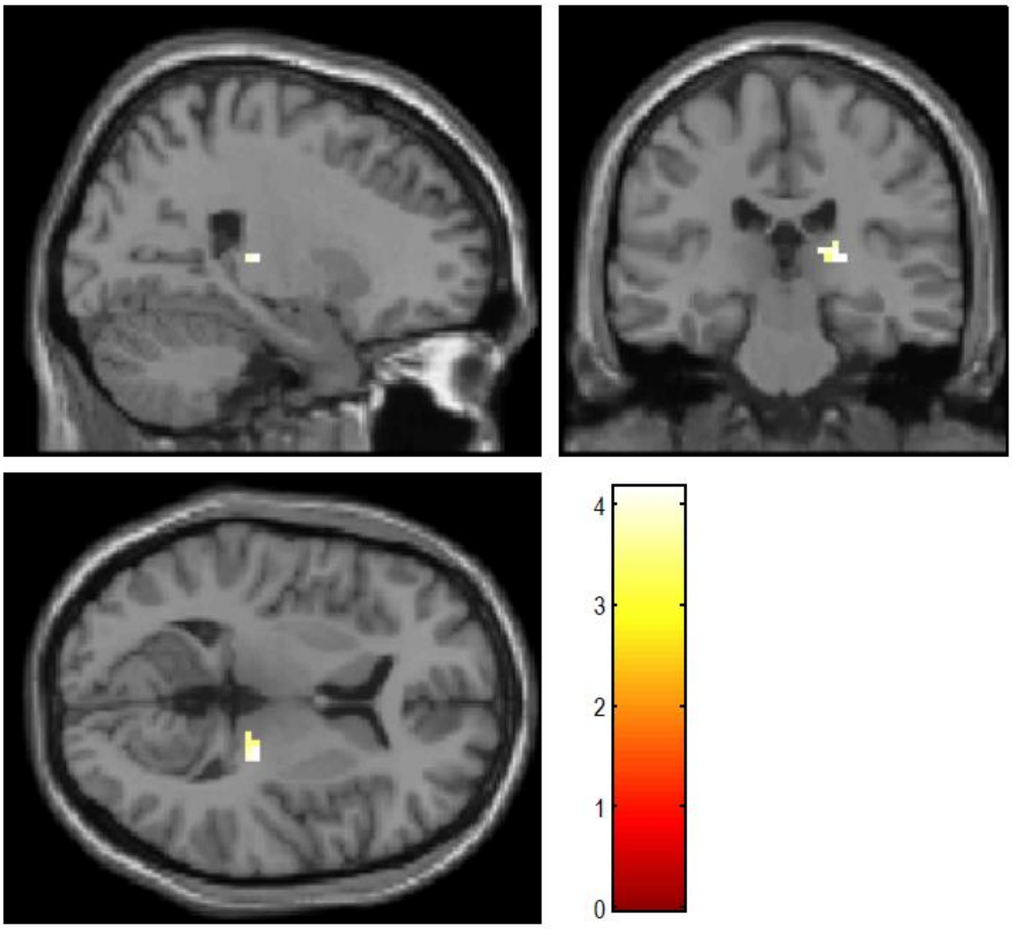

Figure S4. Significantly increased synchronicity between the right mediolateral pulvinar and right amygdala (peak MNI coordinate 24 -25 7, 405 mm^3, Z = 3.75). The figure is presented at p = 0.001. Colour bar indicates T-value.

Figure S5

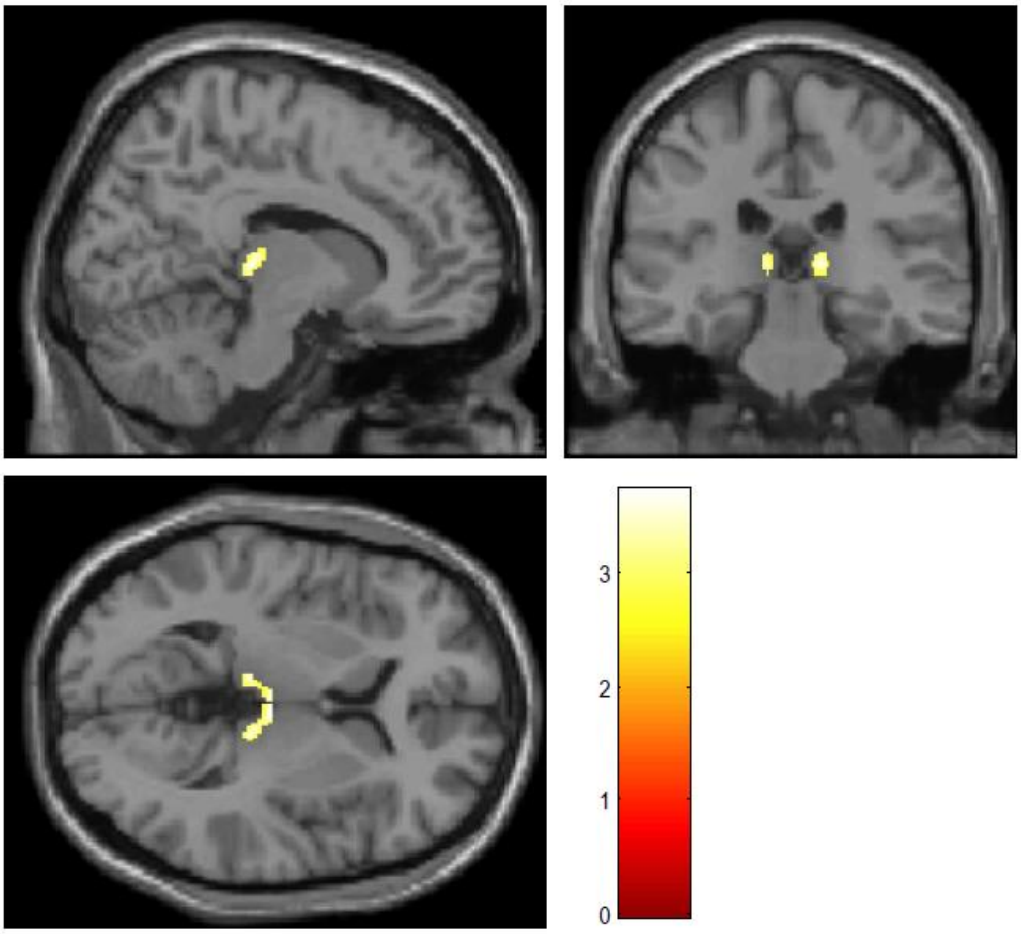

Figure S5. Smaller grey matter volume in the bilateral medial pulvinar with stronger attentional bias toward threat. The figure is shown at MNI coordinate 12 -27 5 with the height threshold $p = 0.005$ for illustration purposes. Colour bar indicates T-value.

References

1. Friston, K. et al. Spatial registration and normalization of images. Human Brain Mapping 2, 165-189. (1995).
2. Ashburner, J. & Friston, K. J. Voxel-based morphometry--the methods. Neuroimage 11, 805-21 (2000).
3. Good, C. D. et al. A voxel-based morphometric study of ageing in 465 normal adult human brains. Neuroimage 14, 21-36 (2001).
4. Ashburner, J. A fast diffeomorphic image registration algorithm. Neuroimage 38, 95-113 (2007).
5. Brett, M. A., JL. Valabregue, R. Poline, JB. in The 8th International Conference on Functional Mapping of the Human Brain (Sendai, Japan., 2002).
6. Morel, A., Magnin, M. & Jeanmonod, D. Multiarchitectonic and stereotactic atlas of the human thalamus. J Comp Neurol 387, 588-630 (1997).
7. Morel, A. The thalamus and behavior: effects of anatomically distinct strokes. Neurology 68, 1640; author reply 1640-1 (2007).
8. Barron, D. S., Eickhoff, S. B., Clos, M. & Fox, P. T. Human pulvinar functional organization and connectivity. Human Brain Mapping 36, 2417-2431 (2015).
9. Etkin, A. & Wager, T. D. Functional Neuroimaging of Anxiety: A Meta-Analysis of EmotionalProcessing in PTSD, Social Anxiety Disorder, and Specific Phobia. American Journal of Psychiatry 164, 1476-1488 (2007).
10. Kuo, J. R., Kaloupek, D. G. & Woodward, S. H. Amygdala volume in combat-exposed veterans with and without posttraumatic stress disorder: A cross-sectional study. Archives of General Psychiatry 69, 1080-1086 (2012).
11. Morey, R. A. et al. Amygdala volume changes in posttraumatic stress disorder in a large case-controlled veterans group. Archives of General Psychiatry 69, 1169-1178 (2012).
12. O'Doherty, D. C. M., Chitty, K. M., Saddiqui, S., Bennett, M. R. & Lagopoulos, J. A systematic review and meta-analysis of magnetic resonance imaging measurement of structural volumes in posttraumatic stress disorder. Psychiatry Research: Neuroimaging 232, 1-33 (2015).
13. Rauch, S. L. et al. Exaggerated amygdala response to masked facial stimuli in posttraumatic stress disorder: a functional MRI study. Biological Psychiatry 47, 769-776 (2000).
14. Krauth, A. et al. A mean three-dimensional atlas of the human thalamus: Generation from multiple histological data. NeuroImage 49, 2053-2062 (2010).
